# Optimizing the automated recognition of individual animals to support population monitoring

**DOI:** 10.1002/ece3.10260

**Published:** 2023-07-03

**Authors:** Tijmen A. de Lorm, Catharine Horswill, Daniella Rabaiotti, Robert M. Ewers, Rosemary J. Groom, Jessica Watermeyer, Rosie Woodroffe

**Affiliations:** ^1^ Department of Life Sciences Imperial College London Silwood Park UK; ^2^ Institute of Zoology Zoological Society of London London UK; ^3^ Division of Biosciences, Department of Genetics, Evolution and Environment, Centre for Biodiversity and Environment Research University College London London UK; ^4^ Department of Zoology University of Cambridge Cambridge UK; ^5^ African Wildlife Conservation Fund Chishakwe Ranch Zimbabwe

**Keywords:** automated individual recognition, Hotspotter, I^3^S‐Pattern, *Lycaon pictus*, photographic identification, WildID

## Abstract

Reliable estimates of population size and demographic rates are central to assessing the status of threatened species. However, obtaining individual‐based demographic rates requires long‐term data, which is often costly and difficult to collect. Photographic data offer an inexpensive, noninvasive method for individual‐based monitoring of species with unique markings, and could therefore increase available demographic data for many species. However, selecting suitable images and identifying individuals from photographic catalogs is prohibitively time‐consuming. Automated identification software can significantly speed up this process. Nevertheless, automated methods for selecting suitable images are lacking, as are studies comparing the performance of the most prominent identification software packages. In this study, we develop a framework that automatically selects images suitable for individual identification, and compare the performance of three commonly used identification software packages; Hotspotter, I^3^S‐Pattern, and WildID. As a case study, we consider the African wild dog, *Lycaon pictus*, a species whose conservation is limited by a lack of cost‐effective large‐scale monitoring. To evaluate intraspecific variation in the performance of software packages, we compare identification accuracy between two populations (in Kenya and Zimbabwe) that have markedly different coat coloration patterns. The process of selecting suitable images was automated using convolutional neural networks that crop individuals from images, filter out unsuitable images, separate left and right flanks, and remove image backgrounds. Hotspotter had the highest image‐matching accuracy for both populations. However, the accuracy was significantly lower for the Kenyan population (62%), compared to the Zimbabwean population (88%). Our automated image preprocessing has immediate application for expanding monitoring based on image matching. However, the difference in accuracy between populations highlights that population‐specific detection rates are likely and may influence certainty in derived statistics. For species such as the African wild dog, where monitoring is both challenging and expensive, automated individual recognition could greatly expand and expedite conservation efforts.

## Graphical abstract

We present a novel framework to automate the individual recognition of animals. Our method automatically selects and crops animals from photo catalogs, and uses photo‐matching software to identify individuals.

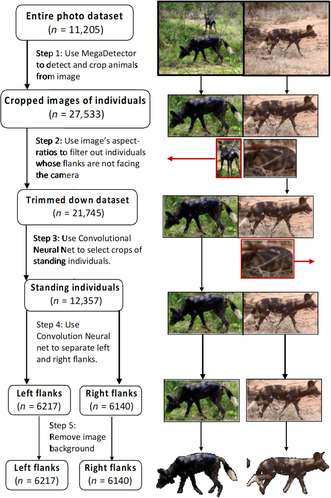

## 1 INTRODUCTION

Reliable estimates of population size and demographic rates are central to monitoring the status of threatened species. However, obtaining individual‐based demographic parameters require long‐term data, gathered through intensive monitoring that is often costly and difficult to conduct (Caughlan, 2001; Horswill et al., 2018). Identification of individuals from photographic records could provide an inexpensive alternative, and open up the possibility of using camera traps and citizen scientists to expand the spatial coverage of monitoring (Marnewick et al., 2014; Seber, 1965; Wearn & Glover‐Kapfer, 2019). This method can be used for species where individuals can be identified from individual markings, including many threatened species (Durant et al., 2014; Pierce & Norman, 2016).

Photographic records have already been used to estimate demographic parameters in several endangered species. For example, long‐term photographic data have been used to obtain survival and abundance estimates of tigers, *Panthera tigris*, and cheetahs, *Acinonyx jubatus*, (Karanth & Nichols, 2011; Kelly et al., 1998), and tourist images have been used to estimate population sizes of whale sharks, *Rhincodon typus* (Davies et al., 2013). In addition, photographs can provide data on individual movement, ranging behavior, and social structure (Armstrong et al., 2019; Randić et al., 2012). Many species are photographed frequently as part of monitoring programs, and by members of the public, including tourists. Such image catalogs therefore represent a large, and potentially underused data resource that inform conservation action.

Nevertheless, visually identifying all individuals in large image databases is time‐consuming. To partly automate this process, several software packages are available to match images based on an individual's unique body markings (e.g., APHIS and WildID, Bolger et al., 2012; Óscar et al., 2015). These image‐matching software packages assist the user by ranking potential image matches using a similarity score. The algorithms underpinning the software packages find these potential matches by comparing images on either a pixel‐by‐pixel or feature basis. Pixel‐based algorithms, such as APHIS, have been successfully applied to numerous species, including horseshoe whip snakes, *Hemorrhois hippocrepis*, and Balearic lizards, *Podiarcis lilfordi* (Óscar et al., 2015; Rotger, 2019). However, they are susceptible to differences in camera angle, scale, and cropping (Matthé et al., 2017), and are therefore unsuitable for animals that cannot be caught and photographed using a standardized methodology. By contrast, feature‐based software packages, such as WildID (Bolger, 2012), I^3^S‐Pattern (Reijns, 2014), and Hotspotter (Crall et al., 2013), match images based on unique features including spots, stripes, blotches, or other marks. The algorithms that feature‐based packages use vary, but all have a higher tolerance to differences in camera angle, scale, and lighting conditions than pixel‐based algorithms.

The feature‐based packages have been tested on a range of taxa (Table 1), and the reported proportion of true matches that the software detects, that is, accuracy rate, varies markedly, ranging between 36% and 100%. This variation can be attributed to differences in species markings, image quality, size of database, how many potential matches were inspected per image, and the image‐matching software used (Crall et al., 2013; Matthé et al., 2017; Nipko et al., 2020). Studies directly comparing the accuracy of different feature‐based packages are considerably more limited, even though the most accurate software differs between species. For example, studies on jaguars, *Panthera onca*, ocelots, *Leopardus pardalis*, and Saimaa ringed seals, *Phoca hispida saimensis*, found that Hotspotter outperformed WildID (Chehrsimin et al., 2018; Nipko et al., 2020), while studies on amphibian species found that WildID outperformed I^3^S‐Pattern (Matthé et al., 2017; Nipko et al., 2020) and Hospotter (Morrison et al., 2016). The only study that directly compared all three software packages found that Hotspotter was superior to I^3^S‐Pattern and WildID for identifying individual green toads, *Bufotes viridis* (Burgstaller et al., 2021). To date, studies comparing image‐matching accuracy across all three software packages for a mammal species are lacking.

**TABLE 1:** A selection of studies that tested the identification accuracy achieved by different image‐matching software packages.

Species	Software package	Accuracy (%)	Number of inspected ranks per image	Reference
Jaguar (*Panthera onca)*	Hotspotter	77	1	Nipko et al. (2020)
Ocelot (*Leopardus pardalis)*		76		
Jaguar	WildID	68		
Ocelot		63		
Thornicroft's giraffe (*Giraffa camelopardalis thornicrofti*)	WildID	71.6	20	Halloran et al. (2015)
Thornicroft's giraffe	WildID	100	1	Bolger et al. (2012)
Leopard cat (*Prionailurus bengalensis*)	Hotspotter	100	1	Park et al. (2019)
Jaguar	Hotspotter	100	1	Crall et al. (2013)
Giraffe (*G. giraffa*)		100		
*Ambystoma opachum*	I^3^S‐Pattern	30.8–48.4	10	Matthé et al. (2017)
	WildID	65.9–82.3		
Yellow‐bellied toad (*Bombina variegata*)	I^3^S‐Pattern	88.6–92.0		
	WildID	96.4–97.3		
Rio grande cooter (*Pseudemys gorzugi*)	I^3^S‐Pattern	41.94	20	Suriyamongkol and Mali (2018)
	WildID	66.13	20	
Green toad (*Bufotes viridis*)	I^3^S‐Pattern	~100	1	Burgstaller et al. (2021)
	WildID	~60		
	Hotspotter	~60		
Wyoming toad (*Anaxyrus baxteri*)	WildID	53	20	Morrison et al. (2016)
	Hotspotter	36		
Italian crested newt (*Triturus carnifex*)	I^3^S‐Pattern	100	5	Sannolo et al. (2016)

Although feature‐based algorithms are better at matching images from different viewpoints than pixel‐based algorithms, researchers are still required to select images that are suitable for identification, in that the distinctive marks must face the camera and must be clearly visible. Furthermore, when these suitable images are selected, the user has to crop the region of interest from the image. For photos that only contain a single animal, this process can be completed in less than 10 s. However, photographs of group‐living animals are likely to contain multiple animals, some of which might be suitable for identification, while others might not. Consequently, manually selecting animals whose marks are clearly visible and then cropping these can take minutes per photo if photos contain a large number of animals. This laborious process is potentially preventing the application of image‐matching software to large image catalogs (Miguel et al., 2019). Parham et al. (2018) automated this preprocessing for giraffes (*Giraffa camelopardalis tippelskirchi* and *G. reticulata*), sea turtles (*Chelonia mydas* and *Eretmochelys imbricata*), humpback whales (*Megaptera novaeangliae*), and zebras (*Equus grevyi* and *E. quaggas*), by using convolutional neural networks (CNNs) to automatically detect these species, putting boundary boxes around individuals, classifying the viewpoint of the image, and partially removing the image background. This work highlights the potential that machine learning methods have for automating this process, although it has only been automated for a few species.

**FIGURE 1:**
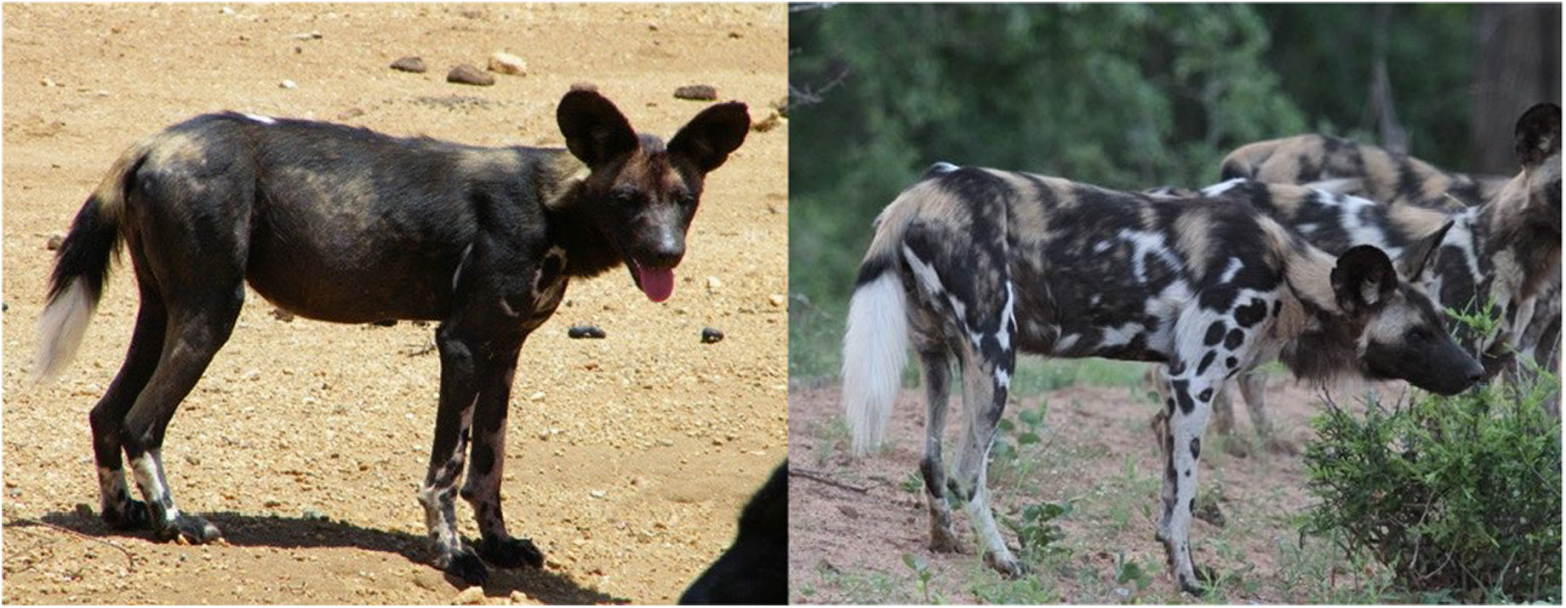
An example of an African wild dog from the Kenyan study site (left), and from the Zimbabwean study site (right).

African wild dogs, *Lycaon pictus*, (hereafter “wild dogs”) have unique coat markings, which vary between individuals (Figure 1, Maddock & Mills, 1994). Wild dogs are classified as globally endangered, and a lack of cost‐effective large‐scale monitoring has been highlighted as a major limitation in developing effective conservation strategies (Woodroffe & Sillero‐Zubiri, 2020). Consequently, there is a pressing need to devise new approaches for monitoring wild dogs. Demographic processes of African wild dogs are typically studied by monitoring a subset of individuals fitted with tracking collars (Jenkins et al., 2015; Rabaiotti & Woodroffe, 2019; Woodroffe et al., 2019). Such collar‐based monitoring is labor‐intensive and expensive, so upscaling is difficult. However, many wild dog packs have already been systematically photographed as part of monitoring programs, and many are also regularly photographed by tourists. Therefore, photographic identification of wild dogs potentially offers a noninvasive, cheaper approach for monitoring, and could reduce uncertainties in demographic rates and expand the spatial representation of monitoring (Maddock & Mills, 1994; Marnewick et al., 2014).

Wild dog coat patterns contain tan, white, and black patches that can vary considerably between populations. For example, wild dogs in eastern African populations tend to have coats consisting of predominantly black fur, while those in southern African populations have relatively more white and tan blotches (Figure 1, McIntosh et al., 2016). In amphibian species with contrasting color patterns of similar blotches, WildID, Hotspotter, and I^3^S‐Pattern have been shown to effectively match images of the same individual, reaching accuracy rates of up to 97% (Burgstaller et al., 2021; Matthé et al., 2017). Therefore, it is likely that feature‐based image‐matching algorithms will effectively identify individual wild dogs from image catalogs. However, variation in the degree of contrast in the color patterns among populations could affect the image‐matching accuracy, and the best performing software package could therefore also vary between populations.

In this study, we develop a method to automatically isolate and crop images from catalogs that are suitable for automated image matching. We then use these images to compare the efficacy of three feature‐based software packages with different underlying image‐matching algorithms (I^3^S‐Pattern, Hotspotter, and WildID; Bolger, 2012; Crall et al., 2013; Reijns, 2014). Finally, we compare whether there is a difference in the accuracy of each software package between two populations with differing coat patterns.

## 2 METHODS

### 2.1 Image datasets

To examine whether the performance of feature‐based image‐matching software packages varies for different populations of African wild dogs, we considered image catalogs from two wild dog populations, one that spans Laikipia, Samburu, and Isiolo Counties in Kenya (37°2′ E, 0°6′ N) and another from the Savé Valley Conservancy in Zimbabwe (32°4′ E, 20°3′ S). The Kenyan dataset contained images taken between 2004 and 2017 (*n* = 9139), and the Zimbabwean dataset contained images taken between 2010 and 2013 (*n* = 2066). In Kenya, these images were taken with 10 different cameras (Olympus© C765UZ, Canon© PowerShot A720IS, EOS Digital Rebel XT, 10D, 40D, 60D, Fujifilm© FinePix S5500, Kodak© Easyshare Z1015IS, Nikon© D70s, and Nikon© Coolpix90). In Zimbabwe, they were taken with five different cameras (Canon© EOS 450D, 20D, Digital Rebel XT, Panasonic© DMC‐FZ20, and Zoran© Coach). Both datasets were collected as part of long‐term monitoring programs, and contained images of both single wild dogs and groups of wild dogs, ranging in their posture from lying down to walking.

### 2.2 Preprocessing steps

**FIGURE 2:**
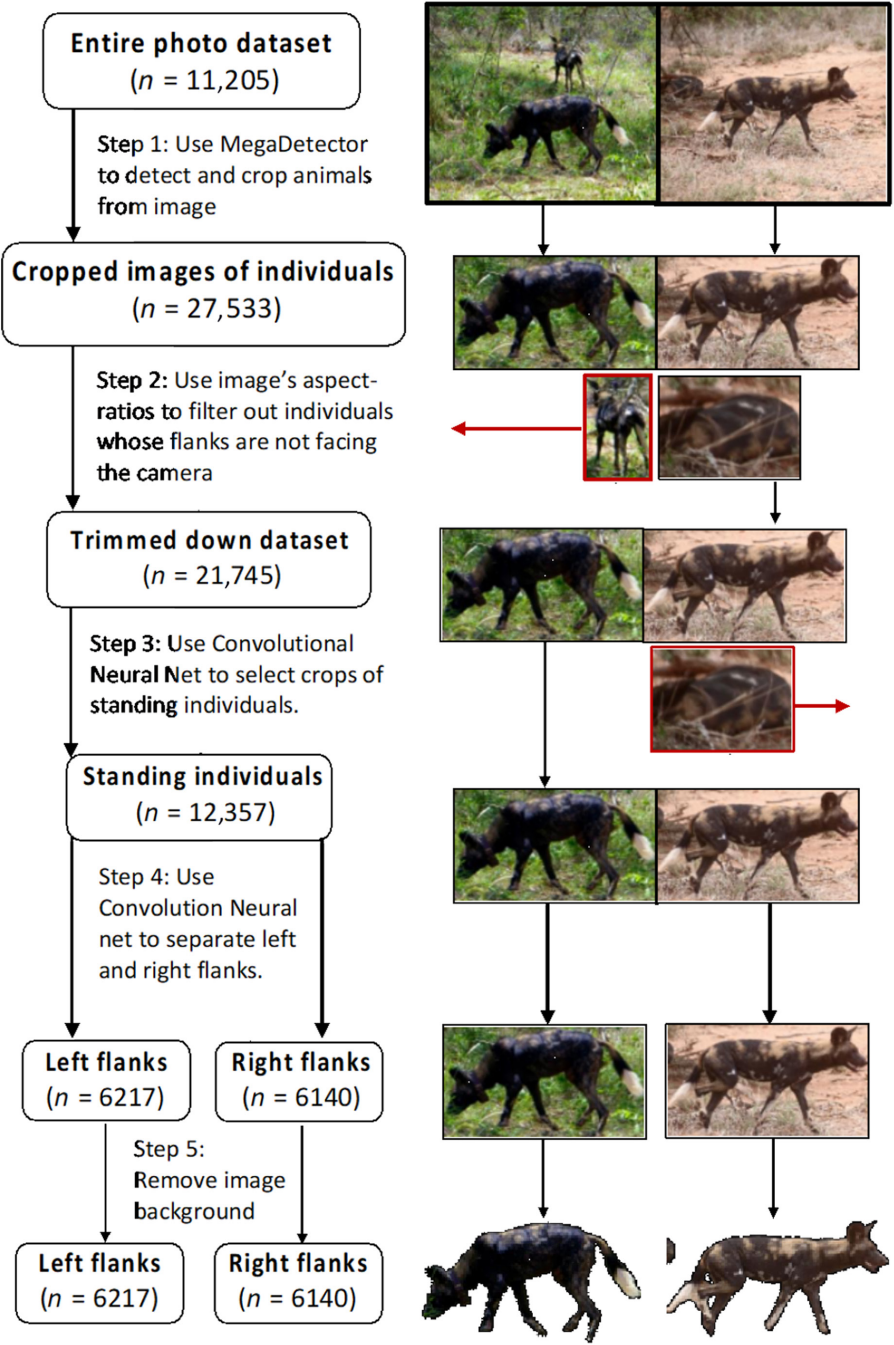
A flowchart describing the image preprocessing steps applied to the combined Zimbabwean and Kenyan image catalog, illustrated using two example images. Images outlined in red are filtered out of the dataset.

To automate the selection of suitable images for image matching, we developed a five‐step image preprocessing method (Figure 2).

#### 2.2.1 Detecting and cropping individuals from images

The aim of the first step in the image preprocessing method was to automatically detect and crop wild dog individuals from the images. To do this, we used the Microsoft AI for Earth MegaDetector (hereafter “MegaDetector”, Beery et al., 2019) that automatically detects and crops animals in images. We assessed the efficacy of this method by visually recording the presence of wild dogs in a subset of 1060 images from the Kenyan dataset and 246 images from the Zimbabwean dataset, and comparing the results to the cropped images (hereafter “crops”) produced by the MegaDetector for the same subset of images. In this way, we obtained the MegaDetector's number of true positives (wild dogs that were successfully detected), false positives (detections which did not contain a wild dog), and false negatives (wild dogs which were found by visual inspection, but not by the MegaDetector). All images contained wild dogs, so there were no true negatives in the dataset.

#### 2.2.2 Aspect‐ratio filtering

The aim of the second step in the image preprocessing method was to filter out images that were unsuitable for identification due to the individual's body rotation in the image, or because of occlusion of the animal's flank by another object or individual. This down‐selection of images ensures that all images depict individuals from roughly the same viewpoint, to maximize the confidence with which images can be matched. We considered crops suitable for image matching if approximately ≥80% of the individual's flank was visible, and the angle between the image axis and animal's flank was less than approximately 30°, that is, the flank was facing the camera. Crops where the angle between the image axis and the animal's flank was more than 30°, and crops where a part of the flank is obscured by another animal were expected to be narrower than crops suitable for image matching and therefore demonstrate a relatively low aspect ratio. By contrast, crops where the flank was concealed because the individual was lying down, or obscured by vegetation, were expected to be considerably wider and demonstrate a relatively high aspect ratio. These criteria were visually assessed for the crops that the MegaDetector produced in the previous step. We then calculated the range of aspect ratios for suitable crops, that is, where an unobscured flank was facing the camera, using the “jpeg” package (Urbanek, 2021) in program R (version 4.0.4, R Core Team, 2020). Images with an aspect ratio outside of this range were removed from the dataset.

#### 2.2.3 Selecting standing individuals

Not all sitting or lying individuals could be filtered out solely using image aspect ratios. Therefore, the aim of the third step in the image preprocessing method was to filter out the remaining crops that were unsuitable for identification because the individual's body position, that is, sitting or lying, obscured the full coat pattern. To do this, we trained a CNN to classify crops as either a standing wild dog or a sitting wild dog. To obtain data to train this image classifier, we used the full image catalogs from both sites (*n* = 11,205). The crops produced by steps 1 and 2 of the preprocessing (*n* = 21,745) were then manually classified as containing either a standing wild dog (*n* = 13,500) or a sitting wild dog (*n* = 6512). We removed all crops depicting anything other than wild dogs (e.g., birds, rocks, or logs), or wild dogs where it could not be confirmed whether they were standing or sitting, because only a small part of the animal was visible (*n* = 1733). We trained a CNN using the remaining 20,012 preprocessed crops, to classify these as containing either a standing wild dog or not. The CNN was made using TensorFlow (Abadi et al., 2016) in Python (Version 3.6.10). The model was trained with 16,012 crops, validated with 2000 crops, and tested with 2000 crops.

CNNs consist of convolutional layers (Albawi et al., 2017): filter layers which digitally “slide” over the image and aim to recognize specific features. The convolutional layers pass a map of specific features to the next layer, a max pooling layer. The max pooling layer reduces the resolution of this feature map, thus reducing the importance of the position of features within this map. This step can help prevent the model from becoming too fine‐tuned to the training data, which causes overfitting and reduces the generalizability of the classifier. After this, a dropout layer is applied, which randomly removes 50% of connections made between layers. This benefits the model by teaching it to recognize robust features, again preventing overfitting. The data are then passed on to a flattening layer, which turns the data into a one‐dimensional string, which is passed onto the final two layers. First, the string goes through a layer which connects all the data from the previous layer and produces prediction scores from the inputs. Second, another layer turns these scores into a single prediction: standing, or not standing (for a more detailed description of CNNs, see Albawi et al., 2017; O'Shea & Nash, 2015).

The number of convolutional layers and the size of the filters that they comprise was optimized using KerasTuner (O'Malley et al., 2019). KerasTuner runs CNNs with a range of values, and automatically selects the model with the highest validation accuracy, that is, the proportion of correct classifications on the validation database. KerasTuner ran CNNs with between one and three convolutional layers, with 16, 32, and 64 filters per layer, and with a kernel size (the number of pixels in the filters) of 3 × 3 pixels. This was done for 20 different random combinations for the number of convolutional layers and number of filters per layer. Test runs showed that the maximum accuracy was reached before the 70th epoch, and therefore each combination was run for 70 epochs, meaning that the training data were passed through the CNN 70 times. The learning rate of the model, that is, the speed at which the model improved itself, was also optimized with KerasTuner, testing a rate of 10^−3^, 10^−4^, and 10^−5^, with the optimal number of convolutional layers. The model with the highest test accuracy was selected as the final model.

#### 2.2.4 Separating left and right flanks

The aim of the fourth step in the image preprocessing method was to separate crops depicting left and right flanks of a wild dog, because image‐matching software packages can only match images for one side of the animal. To do this, we made another CNN to automate the separation of left and right flanks. To obtain training data for this CNN, we visually classified all crops of standing dogs used for the CNN in step three (*n* = 12,357) whose side was facing the camera, as showing the right (*n* = 6140) or left flank (*n* = 6217). We optimized this CNN's parameters as described in step three of the image preprocessing method, using KerasTuner to find the optimal number of convolutional layers and learning rate. Each CNN ran for 100 epochs, because test runs showed that this model took longer than the previous model to reach its maximum accuracy. The first layer of this CNN was an average pooling layer, a layer which reduced the resolution of the input images by a factor of four, which prevents overfitting. This layer was added to this CNN, because preliminary runs showed this CNN was more prone to overfitting than the CNN developed in step three of the image preprocessing method. We used 9857 crops as training data, 1246 as validation data, and 1246 as testing data. All other layers were equal to the previous CNN. For the full model conditions, see Table S1.

#### 2.2.5 Image background removal

Lastly, we removed the image backgrounds of suitable images using the “rembg” package in Python (Gatis, 2020). We removed image backgrounds to remove the risk of the background confounding image‐matching results, while eliminating the need to manually select an individual's flank.

### 2.3 Image‐matching software packages

We compared the performance of three feature‐based image‐matching software packages that differ in the underlying algorithms used to match individuals: I^3^S‐Pattern (Reijns, 2014), WildID (Bolger et al., 2012), and Hotspotter (Crall et al., 2013). All three assist the user by listing potential matches for each image, ranked by a similarity score. The user then confirms which of these potential matches are true matches.

#### 2.3.1 I^3^S‐Pattern

I^3^S‐Pattern uses the Speeded Up Robust Features (SURF) algorithm (Bay et al., 2008; Reijns, 2014) that selects key points and compares each image pair in a dataset based on the size and position of these key points. The software requires the user to select three reference points per image, as well as the outline of the animal. As reference points, we used the base of the tail, the withers (i.e., the ridge between the shoulder blades), and the base of the neck (Figure S1).

#### 2.3.2 WildID

WildID uses the Scale Invariant Feature Transform (SIFT) algorithm (Bolger et al., 2012; Lowe, 2004). It requires the user to input crops of the region of interest: the part of the animal which bears unique marks. The SIFT algorithm detects salient features regardless of their scale and viewpoint. For each image pair in a database, these features are compared, both in how the features look and how the different features are positioned relative to each other. Based on these two characteristics, a goodness‐of‐fit score is computed per image pair.

#### 2.3.3 Hotspotter

Hotspotter also uses the SIFT algorithm to conduct pairwise comparisons (Crall et al., 2013; Lowe, 2004). Users enter either entire pictures of individuals and select the rectangular region of interest, or image crops containing the region of interest. Hotspotter supplements the pairwise comparisons used by WildID with a one‐vs‐many approach that uses a Local Naive Bayes Nearest Neighbor method (McCann & Lowe, 2012) to take all of the images in the database into account when computing similarity scores.

### 2.4 Performance of the image‐matching software

To test which image‐matching software most accurately matched crops of the same individual, we created two separate datasets for the Kenyan and Zimbabwean populations. To select suitable crops, we used the four‐step image preprocessing method described above. We also visually inspected discarded crops to avoid missing suitable crops. We then manually identified individuals from the dataset of right flank crops, to provide a standard against which automated identifications could be compared, and randomly selected two crops per individual. To prevent similar lighting conditions and posture from creating a bias towards matching images of the same individual, we ensured selected images were taken on different days. The two generated datasets consisted of 104 individuals from the Kenyan population and 48 individuals from the Zimbabwean population. To increase the dataset for the Zimbabwean population, we also included left‐flank crops for 41 individuals and horizontally mirrored the crops to enable comparison with the right‐flank crops. This increased the total number of unique flanks from the Zimbabwean population to 89. The coat pattern of wild dogs differs between right and left flanks, and we have no reason to expect that including left‐flank crops would bias our results.

We analyzed the Kenyan dataset with each of the three image‐matching software packages: Hotspotter, WildID, and I^3^S‐Pattern. We then analyzed the Zimbabwean dataset with Hotspotter and WildID. I^3^S‐Pattern was not tested with the Zimbabwean dataset because tests with the Kenyan dataset identified it to be considerably less accurate than the other software packages and considerably more time‐consuming to input images and assign reference points.

We also examined whether image background removal increased the accuracy of WildID and Hotspotter. I^3^S‐Pattern requires users to manually select the outline of the animal in the program and therefore was not included in this analysis, because it does not take the background into account in its default use. We compared the image‐matching results obtained using images from which we manually cropped just the individuals' flanks with those based on crops of complete individuals from which the background was automatically removed (see Figure S2). For three of the 178 images from the Zimbabwe site, the algorithm did not crop out the wild dog, instead cropped out vegetation in the foreground. For these images, a manually cropped flank of the wild dog was used.

To compare the image‐matching performance of each software package, we examined the 10 crops identified as most similar to the sample individual. We used the first 10‐ranked images, as the best performing software's accuracy started leveling off around this rank, indicating that inspecting the first 10 image matches could maximize recognition rates, while minimizing the time spent visually inspecting and confirming potential matches. We used a mixed effects logistic regression to test for differences in the efficacy of the software packages. Here, the response variable was a binary variable describing whether or not an individual was successfully matched in the first 10‐ranked images, and software package was the explanatory variable. Individual identity was included as a random effect to avoid pseudoreplication. Post‐hoc pairwise comparisons were carried out using Tukey contrasts. This analysis was performed separately for the Zimbabwean and Kenyan datasets. Models were run using the “lme4” (v 1.1‐27.1, Bates et al., 2015) package in program R (R Core Team, 2020, version 4.0.4).

Previous studies have shown that the image‐matching performance of different software packages is affected by database size (Matthé et al., 2017). Therefore, to compare software performance on wild dogs from Kenyan and Zimbabwean populations, we randomly selected a subset of the Kenyan individuals to equal the number of identified individuals in the Zimbabwean dataset (*n* = 89). We then used the best performing software package identified in the previous step of the analysis to rerun the image‐matching analysis for both datasets. Differences in software performance between the two populations were then assessed using a mixed effects logistic regression with a binomial link function. The response variable in the model was whether or not a match was detected in the first 10‐ranked images, and study site (Kenya or Zimbabwe) was the explanatory variable. To correct for possible differences in image quality, two proxies for image quality were included in the model. First, we included image size (total number of pixels of the crop) as a continuous predictor. Second, all images were visually scored on a scale of 1–3, based on how well their distinct marks could be recognized. This approach followed Nipko et al. (2020), where score 1 was given to images that were out of focus, of a moving animal, or badly lit, score 2 was given to images of intermediate quality, and score 3 was given to images where all features were clearly visible (e.g., see Figure S3). Score was included as a fixed effect and individual identity was included as a random effect. Furthermore, a Wilcoxon rank sum test was performed to test for differences between the quality score of crops from Kenya and Zimbabwe. The model was fit using the “lme4” package (v 1.1‐27.1, Bates et al., 2015) in R (version 4.0.4, R Core Team, 2020).

## 3 RESULTS

### 3.1 Automatic detection and aspect ratio filtering

The Microsoft AI for Earth MegaDetector, which is designed to automatically detect and crop animals from images, produced 2652 crops from the test dataset of 1306 images, meaning that on average, approximately two detections were made per image. Of these, 2523 crops contained a wild dog (true positive rate = 0.951), 129 were false detections, such as rocks or vegetation (false positive rate = 0.049), while 531 wild dogs were not successfully detected (false negative rate = 0.174). However, only five of these false negatives were images suitable for image matching. By contrast, the flank of the wild dog was not visible in the other 526 false negatives. In total, 722 crops were suitable for identification using image‐matching software, of which five were not detected by the automated processing (false negative rate = 0.007). For the 2652 crops that were produced by the MegaDetector, all crops considered suitable for identification on visual inspection had an aspect ratio between 0.65 and 2.25. Applying the MegaDetector to the entire image dataset (*n* = 11,205), as opposed to the test dataset, resulted in 21,745 crops. Of these, 5788 (21%) fell outside the suitable range of aspect ratios and were therefore removed from the dataset.

### 3.2 Using convolutional neural nets to filter out unsuitable images

The optimal conditions for the CNN trained to recognize wild dogs standing up were two convolutional layers with 32 and 64 filters, respectively, and a learning rate of 10^−5^. This model achieved a training accuracy of 100%, a validation accuracy of 91% (95% CI 90–92), and a testing accuracy of 90% (95% CI 88–91, Table 2). For the CNN designed to separate images of the left and right flanks, the optimal conditions were three convolutional layers, one with 64 filters and two with 32 filters, with a learning rate of 10^−4^. Its training, validation, and testing accuracy were 100%, 96% (95% CI 95–97), and 95% (95% CI 94–96), respectively.

**TABLE 2:** The accuracy and 95% confidence intervals of the best performing Convolution Neural Networks aiming to classify images of African wild dog into (1) those depicting an individual standing up and not standing up and (2) those depicting left or right flanks.

Model	Training accuracy (%)	Validation accuracy (%)	Testing accuracy (%)
Standing/not standing classifier	100	91 (90–92)	90 (88–91)
Left/right flank classifier	100	96 (94–97)	95 (94–96)

### 3.3 Performance of the image‐matching software packages

**FIGURE 3:**
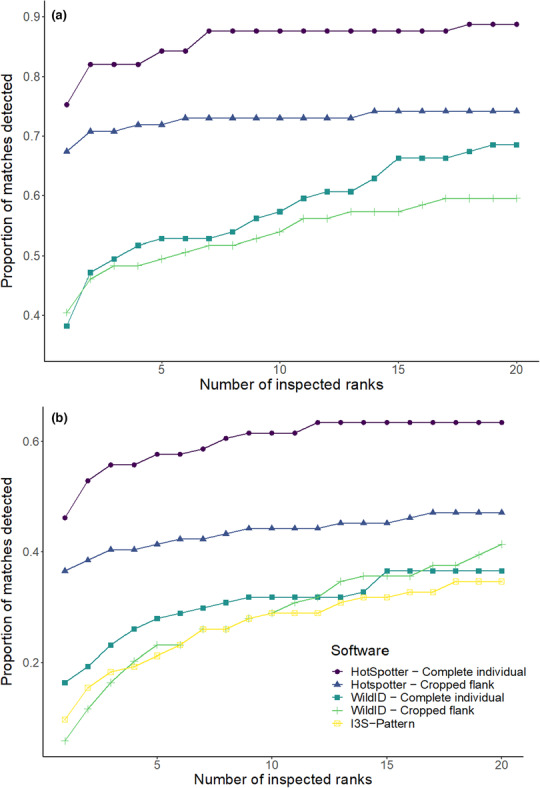
The proportion of true matches in the dataset that were detected within the top 20‐ranked images using different software packages for (a) 89 Zimbabwean and (b) 104 Kenyan African wild dogs, that were visually matched as a reference.

For both the Kenyan and Zimbabwean datasets, Hotspotter achieved the highest image‐matching accuracy (Figure 3). For the Kenyan dataset, using Hotspotter with crops of the full individual from which the background was removed, was most effective. This method detected 62% of the matches in the 10 highest ranked crops (Figure 3b). This was significantly higher than using manually cropped flanks of the individual in both WildID (*z* = 5.0, *p* < .01) and Hotspotter (*z* = 2.8, *p* = .046), as well as crops of the full individual in WildID (*z* = 4.7, *p* < .01) and I^3^S‐Pattern (*z* = 5.0, *p* < .01).For the Zimbabwean dataset, Hotspotter detected 88% of matches within the first 10‐ranked images when the background was removed from crops of the full individual (Figure 3a). The matching performance was significantly lower when crops of just the flank were used (*z* = 2.7, *p* = .03). Hotspotter with background removal performed significantly better than WildID with background removal, (*z* = 4.7, *p* < .01), and WildID with crops of the flanks (*z* = 5.1, *p* < .01).

**FIGURE 4:**
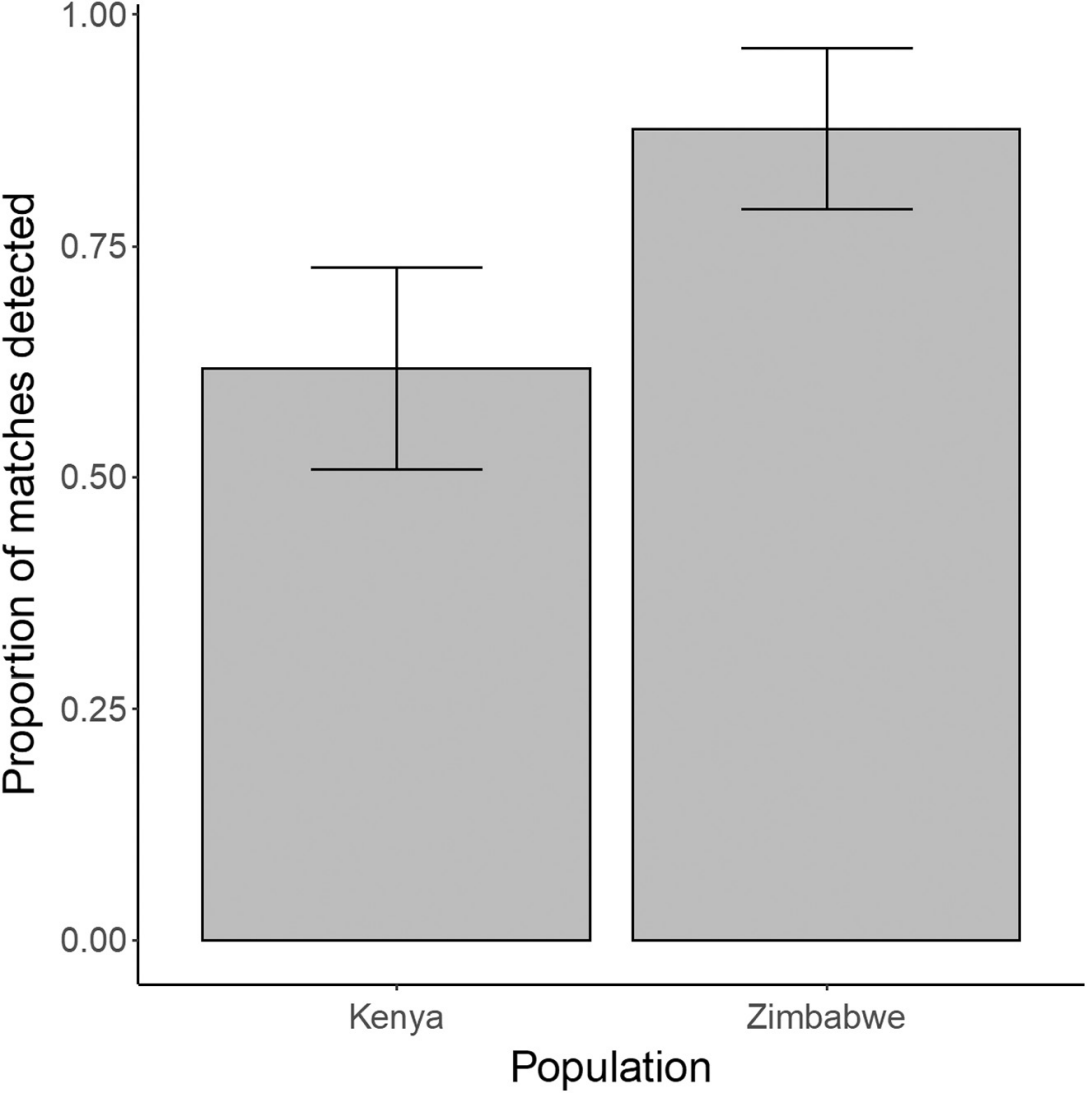
The proportion of image matches detected within the 10 highest ranked images by Hotspotter for the Zimbabwean and Kenyan populations (*n* = 89 individuals for both populations). Error bars denote 95% confidence intervals.

The probability of accurate image matching occurring within the first 10‐ranked images was significantly higher for wild dogs from Zimbabwe than for wild dogs from Kenya (OR = 9.64, 95% CI 3.65–15.63, Figure 4). The proportion of matched individuals identified in this analysis was not significantly associated with image size (*X* ^2^_1_ = 0.16, *p* = .69) or image quality (OR_Quality Score 2/Quality Score 1_ = 0.89, 95% CI −2.26 to 4.04, OR_Quality Score 3/Quality Score 1_ = 1.82, 95% CI −2.20 to 5.83). In addition, the image quality score did not differ between the populations (*W* = 15,008, *p* = .33).

## 4 DISCUSSION

This study presents a novel framework for automating the individual recognition of species with distinct marks. The framework includes an automated preprocessing method for identifying images suitable for image matching, and then using image‐matching software for individual recognition. The automated preprocessing method consists of five steps that (1) crop all images containing animals from a large database, (2) filter out a portion of the unsuitable images based on image aspect ratio, (3) use CNNs to select images of standing individuals (accuracy of 90%), (4) separate images into left and right flanks (accuracy of 95%), and (5) remove image backgrounds. As a case study, we applied the described methods to an image catalog of African wild dogs and found that Hotspotter (Crall et al., 2013) was the most efficient software package for matching images. Image‐matching performance was also significantly improved by using the full image of an individual from which the background was removed, as opposed to just the cropped flank. Finally, we found that image‐matching performance differed between populations of wild dogs with different coat coloration patterns. This work showed that image‐matching software could become a powerful method for monitoring populations of African wild dogs. However, caution is needed as detection rates are likely to vary between—and even within—populations. This could affect the certainty of derived population‐specific demographic parameters, such that careful consideration is needed to account for individual heterogeneity in detection when large variation in coat coloration occurs within a population.

The automated preprocessing method presented in this study could eliminate the need to manually select suitable images for image matching and crop individuals from original photographs. This method thus enables processing of large image catalogs where selection using visual inspection would be extremely time‐consuming. We found that the method does discard a small number of suitable images, and therefore in situations where it is important to include all suitable images, the preprocessing method outlined here could also be used as a presorting approach. The user could then visually review images that were classified as not suitable, to prevent usable images from being discarded.

The described method of preprocessing is particularly useful for wild dogs, since an individual's posture varies substantially between images. Images taken by tourists provide an opportunity to bolster and spatially extend image catalogs. However, these images are also likely to contain many images unsuitable for identification, as they are not taken for the purpose of identification. Accordingly, filtering unsuitable images from these datasets using an automated approach could be especially timesaving. The described preprocessing method is therefore highly suitable to species targeted by wildlife‐watching excursions, that have distinctive marks and where individual posture influences image suitability, for example, cheetahs, leopards (*Panthera pardus*), and tigers.

Hotspotter outperformed I^3^S‐Pattern and WildID at matching images of individual wild dogs. This finding agrees with studies on green toads that compared Hotspotter and I^3^S‐Pattern (Burgstaller et al., 2021), as well as studies comparing Hotspotter and WildID (Burgstaller et al., 2021; Chehrsimin et al., 2018; Nipko et al., 2020). Nevertheless, this result is not ubiquitous. WildID was superior to Hotspotter at matching images for a blotched amphibian species, the Wyoming toad, *Anaxyrus baxteri* (Morrison et al., 2016). This indicates that the identification performance of different software packages is dependent on species, even when two species' patterns show similarities. Consequently, we recommend that all three software packages are tested on new species before deciding on which one to use.

Using crops of full individuals from which the background was removed significantly increased the image‐matching accuracy of Hotspotter, compared to using crops of just individuals' flanks. This method also speeds up image preprocessing by eliminating the need to manually crop the region of interest. The improved accuracy is likely caused by two factors. First, removing the background prevents images being matched based on similar backgrounds, as the flanks are not perfect rectangles, meaning that crops of the flank also contain some background (see Figure S2). Second, using complete individuals allow images to be matched based on unique features on the legs, in addition to the flanks. This result is in line with studies on Saimaa ringed seals, *Pusa hispida*, and Thornicroft's giraffes, *G. camelopardalis thornicrofti*, which found evidence that using a full individual from which the background is removed, could result in a higher accuracy (Chehrsimin et al.,  2018; Halloran et al., 2015). However, neither of these previous studies statistically tested whether background removal increased identification accuracy. Our study therefore provides the first statistical evidence that background removal can increase the performance of image‐matching software. This also indicates that the common usage of Hotspotter, in which a rectangular region of interest is manually cropped (e.g., Dunbar et al., 2021; Nipko et al., 2020), could be improved by removing the image background.

Hotspotter was significantly better at matching images from Zimbabwean wild dogs, compared to Kenyan individuals. The higher image‐matching accuracy found for the Zimbabwean population is likely to reflect the regional difference in wild dog coat coloration patterns. The Kenyan population has darker, more uniform coats, consisting of large black patches, often with few white or tan areas (Daniels et al., 2022; McIntosh et al., 2016). By contrast, the proportion of tan fur is ~1.5 times higher, and the proportion of white fur is almost seven times higher for the Zimbabwean population (Figure 1, Daniels et al., 2022). Therefore, the higher contrast within the patterns of the Zimbabwean wild dogs could make it easier for the software to match images of these individuals. The identified relationship between image‐matching performance and software package remained unaltered when image quality and image size were included in analyses, and there was no significant difference between the image quality scores between the Zimbabwean and Kenyan populations. The image quality score approach was modeled after Nipko et al. (2020), who found that it significantly affected the probability of matching ocelot and jaguar individuals. As a result, we are confident that the differences in coat coloration patterns between wild dogs from Zimbabwe and Kenya reflect variation in identification performance between populations.

Interpopulation variation in image‐matching performance indicates that detection probabilities derived from using this approach will not be directly comparable between populations. Since the probability of finding an accurate image match depends on individual coat pattern, this finding highlights that individual heterogeneity in detection may also occur if large variation in coat coloration occurs within a population. Capture‐mark‐recapture techniques assume individuals' experience equal detection probability across a population (White & Burnham, 2009). Therefore, individual coat pattern may also need accounting for when deriving survival estimates using such analysis. This also applies to other species whose coat pattern varies regionally, such as Asian golden cats, *Catopuma temminckii*, and ocelots (Allen et al., 2011; Khan et al., 2017). Furthermore, the coat patterns of other wild dog populations can differ considerably from the two populations included in this study (Daniels et al., 2022, McIntosh et al., 2016). Consequently, we advocate that estimating a population‐specific image‐matching accuracy score becomes an essential prerequisite step for applying these techniques in different locations.

Automatically preprocessing wild dog image datasets and using image‐matching software facilitates the use of archived and citizen science image catalogs where visually identifying all individuals would be extremely time‐consuming. Although the best performing image‐matching software did not detect all matches, it could be used to identify a large proportion of the individuals in a dataset. Afterwards, individuals that were not matched to any other images could be visually identified, to prevent missing actual matches. Using image‐matching software in this way still saves time by rapidly identifying a large portion of the matches, without compromising on accuracy. Furthermore, it is plausible that the likelihood of correctly detecting matching images increases if more than two images per individual are included, for example, if multiple viewpoints per individual are present in a dataset, the probability of matching these is expected to increase (Crall et al., 2013). Our accuracy values therefore represent a conservative estimate of Hotspotter's true accuracy.

Our study indicates that image matching could provide a valuable new approach for monitoring wild dogs. A combination of citizen science and image matching has already been successfully employed to monitor other species, such as Blanding's turtles, *Emydoidea blandingii*, and whale sharks (Araujo et al., 2017; Cross et al., 2021). Similarly, previous studies have used tourist images to estimate the population size of wild dogs in Kruger National Park, South Africa (Marnewick et al., 2014). Combining citizen science, image‐matching software, and capture–recapture methods therefore has the potential to improve the understanding of wild dog demography. However, more research is needed to investigate whether photographic data could improve our understanding of wild dog demography beyond population size, by estimating parameters such as pack structure, dispersal rates, and death and birth rates. This can be achieved by applying image‐matching software to existing image datasets, to assess whether they generate enough data to estimate key demographic parameters, or whether more intensive monitoring—for example, using long‐term camera trap surveys—would be necessary.

In conclusion, we have developed a new automated method for preprocessing image datasets, by automatically cropping animals from images, removing images in which the individuals' posture hinders identification, separating left and right flanks, and removing the image background. This framework will enable large image datasets to be analyzed rapidly, thereby expanding monitoring efforts and expediting conservation action. Furthermore, we have shown how well different image‐matching software packages perform on African wild dogs. Hotspotter outperformed the other software packages, while its performance differed between two populations which exhibit intraspecific variation in their coat patterns. Our preprocessing method, in combination with Hotspotter, has immediate application in research and monitoring efforts for wild dogs and other species. Data obtained in this way could provide cost‐effective large‐scale monitoring for endangered species, therefore supporting the implementation of effective conservation.

## AUTHOR CONTRIBUTIONS

**Tijmen A. de Lorm:** Conceptualization (equal); data curation (equal); formal analysis (lead); methodology (equal); writing – original draft (lead); writing – review and editing (lead). **Catharine Horswill:** Conceptualization (equal); methodology (equal); supervision (equal); writing – original draft (equal); writing – review and editing (equal). **Daniella Rabaiotti:** Conceptualization (equal); methodology (equal); supervision (equal); writing – original draft (equal); writing – review and editing (equal). **Robert M Ewers:** Conceptualization (equal); methodology (equal); supervision (equal); writing – original draft (equal); writing – review and editing (equal). **Rosemary J Groom:** Data curation (equal); writing – original draft (equal); writing – review and editing (equal). **Jessica Watermeyer:** Data curation (equal); writing – original draft (equal); writing – review and editing (equal). **Rosie Woodroffe:** Conceptualization (equal); data curation (equal); methodology (equal); supervision (equal); writing – original draft (equal); writing – review and editing (equal).

## Supporting information


Appendix S1

## Data Availability

The code used for data analysis and image preprocessing, and all results, are available on Zenodo: https://zenodo.org/record/7712853#.ZAnrSy‐l0Vw. The full image datasets are available on request.
