# Correction to “Optimizing the automated recognition of individual animals to support population monitoring”

**DOI:** 10.1002/ece3.10510

**Published:** 2023-09-12

de Lorm, T. A., Horswill, C., Rabaiotti, D., Ewers, R. M., Groom, R. J., Watermeyer, J., & Woodroffe, R. (2023). Optimizing the automated recognition of individual animals to support population monitoring. *Ecology and Evolution, 13*, e10260. https://doi.org/10.1002/ece3.10260

In table 1, the cited accuracies of Burgstaller *et al*.'s 2021 study on green toads (*Bufotes viridis*) are incorrect. The correct values are ~100 for Hotspotter, ~60 for WildID, and ~40 for I3S‐Pattern.

We apologize for this error.

