# Supplementary material for: Optimizing the automated recognition of individual animals to support population monitoring
Source: Ecol Evol. 2023 Jul 3;13(7):e10260. doi: 10.1002/ece3.10260 (PMC10316465; doi:10.1002/ece3.10260)
Supplement: Supplementary file 1 — Appendix S1 [file ECE3-13-e10260-s001.docx]

**Supplementary Information for**

**Optimising the automated recognition of individual animals to support population monitoring**

**T.A. de Lorm, C. Horswill, D. Rabaiotti, R.M. Ewers, R. J. Groom, J. Watermeyer, R. Woodroffe**

**Table S1** The structure of a Convolutional Neural Net that classifies images of African Wild Dogs into “standing” or “not standing” (S1.A), and a Convolutional Neural Net that classifies images of African Wild Dogs into “left flank” or “right flank” (S1.B). The layers are given in the order at which they occur in the model. The models were optimised using RMSprop, an algorithm which guides how the model improves itself (Tieleman & Hinton, 2012). The activation column refers to which activation function was used in each layer, which determines how the nodes within layers convert its input to an output-value. ReLu was used as activator function, as this has been found to improve multi-layer networks (Glorot, Bordes & Bengio, 2011). The last layer is activated with a Sigmoid function, which turns the input into a single, binary prediction (“standing” or “not standing”, “left” or “right”).

**S1.A**

| **Layer** | **Size** | **Activation** |
| --- | --- | --- |
| **Convolutional** | 32 filters | Rectified Linear Activator (ReLu) (Agarap, 2019) |
| **Convolutional** | 64 filters | ReLu |
| **Max Pooling 2D** | NA | NA |
| **Dropout** | NA | NA |
| **Flatten** | NA | NA |
| **Dense** | 128 neurons | ReLu |
| **Dense** | 1 | Sigmoid |

**S1.B**

| **Layer** | **Size** | **Activation** |
| --- | --- | --- |
| **Average Pooling 2D** | NA | NA |
| **Convolutional** | 32 filters | ReLu |
| **Convolutional** | 64 filters | ReLu |
| **Convolutional** | 64 | ReLu |
| **Max Pooling 2D** | NA | NA |
| **Dropout** | NA | NA |
| **Flatten** | NA | NA |
| **Dense** | 128 neurons | ReLu |
| **Dense** | 1 | Sigmoid |

**
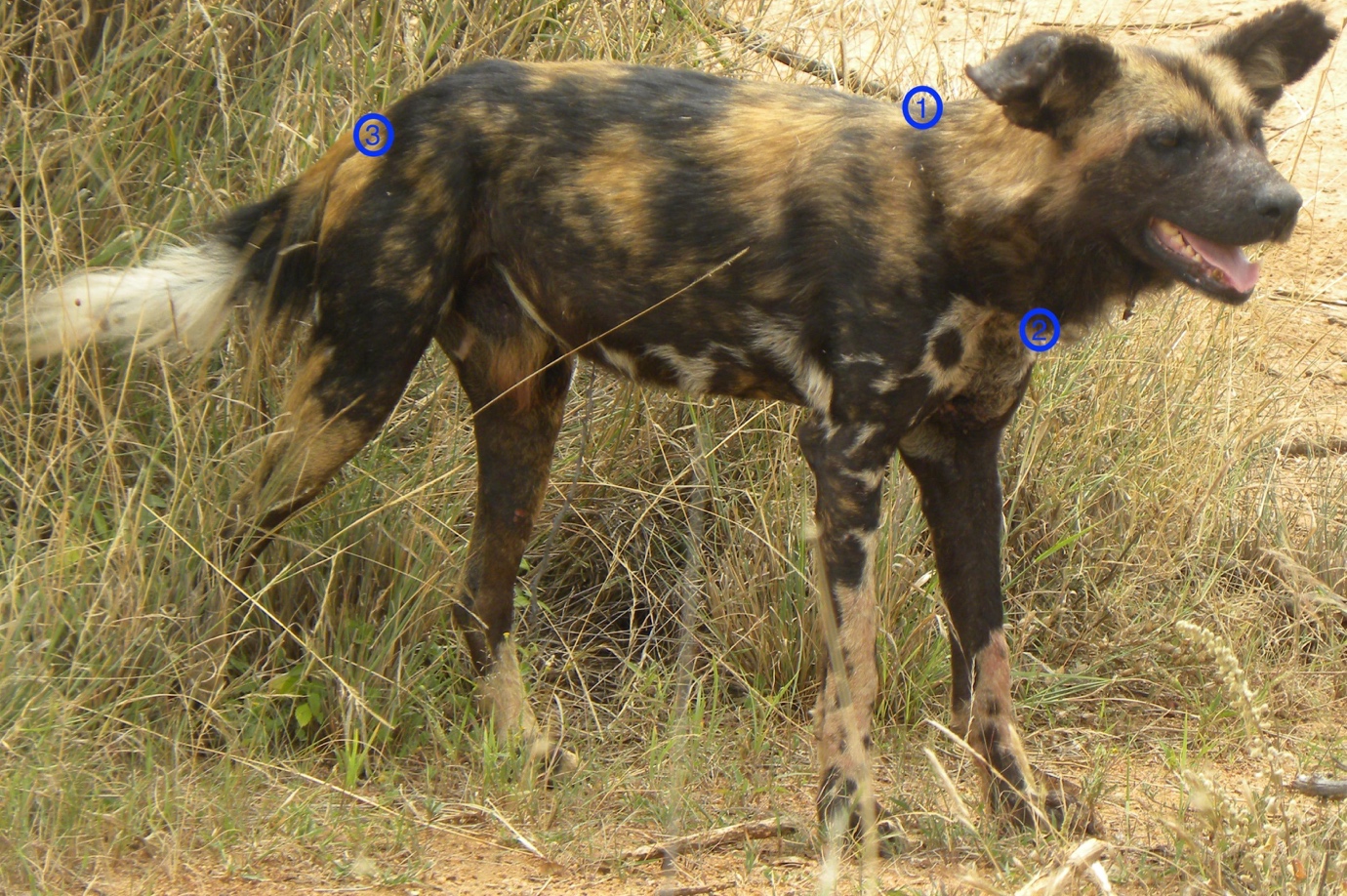
**

**Figure S1.** An image with the reference points used for I^3^S-Pattern: (1) the withers, (2) the base of the neck, and (3) the base of the tail.


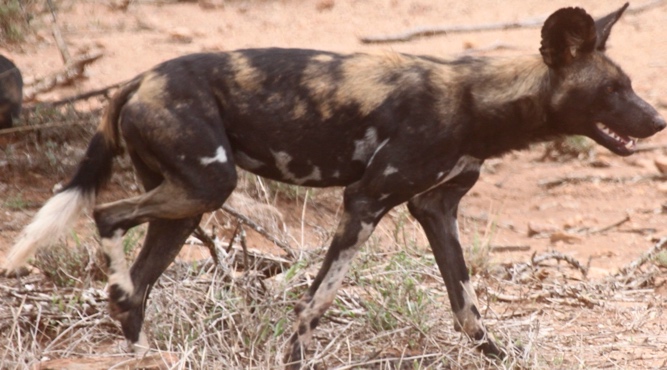

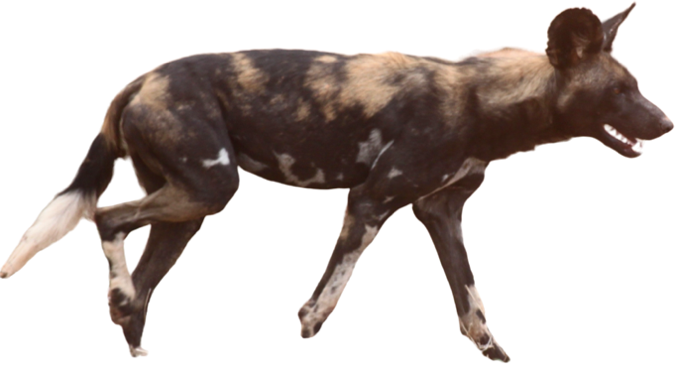


**Figure S2.** The two ways in which WildID and Hotspotter were tested: with crops of the full individual from which the background was removed (left), and manually cropped flanks of the individual (right).

**Score = 1**

**Score = 2**

**Score = 3**

**Figure S3.** Three images representative of the image quality scores that were assigned to images included in a Mixed Effect Logistic Regression. The regression tested whether there was a difference in the accuracy of Hotspotter between the Kenyan and Zimbabwean population. The image quality score was included to correct for any potential influence of image quality.

## References

Glorot, X., Bordes, A. & Bengio, Y. (2011) Deep Sparse Rectifier Neural Networks. In: Proceedings of the Fourteenth International Conference on Artificial Intelligence and Statistics. 14 June 2011 JMLR Workshop and Conference Proceedings. pp. 315–323. <https://proceedings.mlr.press/v15/glorot11a.html>.

Tieleman, T. & Hinton, E. (2012) *Lecture 6.5 - rmsprop, COURSERA: Neural networks for machine learning*.
